# Effect of gasoline additive on combustion and emission characteristics of an n-butanol Partially Premixed Compression Ignition engine under different parameters

**DOI:** 10.1038/s41598-021-81490-3

**Published:** 2021-01-21

**Authors:** An Lu, Chunhua Zhang, Peng Ji, Yangyang Li

**Affiliations:** School of Automobile, Chang’an University, Xi’an, 710064 People’s Republic of China

**Keywords:** Engineering, Energy science and technology, Renewable energy

## Abstract

The experiments were conducted on a modified two-cylinder diesel engine to investigate the effects of excess-air coefficient (*λ*) and intake temperature (*T*_*in*_) of different blending ratios (volume ratio of gasoline in the blends) on the combustion and emission characteristics of a Partially Premixed Compression Ignition (PPCI) engine. The results show that with the increase of gasoline blending ratio, the peak in-cylinder pressure (*P*_*max*_), the peak in-cylinder temperature (*T*_*max*_) and the peak heat release rate (*HRR*_*max*_) of four test fuels all increase first and then decrease. When gasoline volume fraction is 10%, HC and CO emissions are the lowest. In addition, intake temperature (*T*_*in*_) has a significant effect on the n-butanol/gasoline PPCI engine. With the increase of *T*_*in*_, the in-cylinder *P*_*max*_ and *HRR*_*max*_ of four test fuels gradually increase, the combustion phase advances and HC and CO emissions decrease, while NOx emissions increase slightly. Furthermore, as *λ* increases, the *P*_*max*_, *T*_*max*_ and *HRR*_*max*_ of the four test fuels show monotonously reducing trend. At the same time, mixture concentration has basically no effect on start of combustion (*CA10*), the combustion duration (*CD*) gradually extends, and HC and CO emissions increase.

## Introduction

Energy and environmental issues are the focus of attention in today's world. The internal combustion engine, as the main source of energy consumption, is a popular target for researchers. At the same time, environmental pollution caused by internal combustion engine emissions is also a hot spot for countries all over the world. Today's higher fuel economy requirements and stricter emission regulations pose great challenges to traditional combustion methods. Homogeneous Charge Compression Ignition (HCCI) as a new type of combustion method can greatly improve thermal efficiency and obtain lower NOx and soot emissions, but HCCI also faces problems such as uncontrollable combustion phase, narrow operating range, and difficulty in practical application^1–3^. At present, most researchers believe that HCCI combustion is mainly controlled by fuel chemical kinetic reaction, so its combustion phase is difficult to control. On the other hand, higher pressure rise rate may also result in greater combustion noise. In addition, high CO and HC emissions and cold start problem are also challenges faced by HCCI^3^. Similar to HCCI combustion are Partially Premixed Compression Ignition (PPCI), Active Thermo-Atmosphere Combustion (ATAC) and Reactivity Controlled Compression Ignition (RCCI).

Kalghatgi et al.^4^ proposed PPCI from the perspective of improving the premixed combustion of diesel engines, using a single injection of gasoline in compression stroke to achieve fuel stratification. PPCI requires that the injection must end before the combustion starts, so it is a kind of partial premixed combustion. Weall et al.^5^ used a single injection strategy to achieve gasoline/diesel (50%/50%) PPCI combustion. Under the condition of medium load 0.7 MPa BMEP and 1600 r/min, they found that NOx, soot, CO and HC emissions were 0.41, 0.032, 4.94 and 0.65 g/(kW·h), respectively. Such low emissions results cannot be achieved with pure diesel. In addition, they compared the results of two injection pressures (100 MPa and 150 MPa) and found that the dependence of soot emission on injection pressure was greatly reduced with high degree of premixing blends. This is consistent with the results of literature^6^. Han et al.^7^ also pointed out that the injection pressure might affect mixture concentration and ignition delay, which in turn affected HC and CO emissions. Kim et al.^8^ conducted a multiple premixed compression ignition (MPCI) load expansion test of naphtha on a compression ignition engine. They found that by optimizing injection parameters and intake control, the indicated mean effective pressure (IMEP) of naphtha MPCI mode could reach 1.4 MPa, which had high thermal efficiency, low emissions and low combustion noise. However, it is difficult to achieve MPCI combined combustion by using two-stage heat release under low load conditions. This is because combustion duration is too long, resulting in unstable combustion. At this time, the thermal efficiency and combustion stability of the PPCI mode are better.

In order to expand fuel compression ignition load range and try to ensure premixed combustion, researchers have tried in many ways. Premixed compression ignition is mainly affected by three factors: fuel ignitability, concentration and temperature. Therefore, fuel characteristics and engine intake temperature have important influence on PPCI combustion. Najt et al.^9^ confirmed that using intake heating and external EGR could achieve spontaneous ignition of isooctane and gasoline combustion. Ibrahim et al.^10^ studied the influence of intake temperature on hydrogen engines, and found that combustion stage was significantly advanced with the increase of intake temperature. In addition, it was also found that wall temperature, engine speed and intake strategy could affect the thermal stratification in the combustion chamber^11,12^. For the study of fuel characteristics, researchers have paid close attention to the impact of fuel cetane number and octane number on combustion and emissions. Wang et al.^13^ studied the combustion and emission characteristics of naphtha, gasoline/n-heptane blends, and gasoline/diesel blends in MPCI mode. The results showed that pressure rise rate, soot and NO emissions of diesel fuel were much higher than gasoline. Therefore, in terms of emissions, gasoline-type fuels are more suitable for compression ignition engines than diesel.

For the combination of different fuel types and combustion modes, Vallinayagam et al.^14^ compared the combustion and emission characteristics of naphtha and diesel/gasoline blends in different combustion modes. They found that for dieseline fuel to obtain the same combustion phase as naphtha, HCCI mode required higher intake temperature, and CI mode required lower intake temperature. However, under partially premixed combustion (PPC) conditions, all test fuels required almost the same intake temperature to obtain the same combustion phase as naphtha. Analysis showed that the stratified combustion of dieseline was more obvious, while the combustion of naphtha and PRF60 was premixed combustion.

As the second-generation biofuel, n-butanol has good explosion resistance and high energy density^15,16^, its low calorific value is higher than that of ethanol, and its water absorption and corrosivity are relatively low. Butanol is more miscible with gasoline. Therefore, the application of n-butanol on HCCI engines has broad prospects. Liu et al.^17^ studied the combustion characteristics of n-butanol HCCI. They found that air dilution and reducing the effective compression ratio could effectively reduce the maximum pressure rise rate and delay the ignition time of n-butanol. He et al.^18^ studied the combustion and emission characteristics of an HCCI engine fueled with n-butanol/gasoline blends. They found that an increase in proportion of n-butanol could advance the ignition time and shorten the combustion duration. Studies^19,20^ have shown that controlling the injection time in the cylinder can directly affect the time scale of chemical reaction of combustion, so as to achieve effective control of ignition time. Li et al.^21^ conducted numerical simulations on the combustion characteristics of methanol/diesel RCCI engines. The results showed that increasing the proportion of methanol was beneficial to improve fuel economy and had a positive effect on avoiding engine knock. Zheng et al.^22^ conducted a single-cylinder n-butanol HCCI engine performance study on a modified four-cylinder diesel engine, and compared the test results with the benchmark diesel HCCI. The results showed that the lower reactivity of n-butanol helped to achieve the best combustion phase after top dead center (TDC), and had a thermal efficiency equivalent to that of traditional diesel engines (43–46%). Compared with diesel HCCI, load range of n-butanol HCCI could be extended to 10 bar IMEP (up to 25% higher than diesel HCCI), while maintaining ultra-low NOx and soot emissions, and had better engine performance than diesel HCCI. However, when the concentration of n-butanol mixture was low, the misfire frequency of the HCCI engine increased, and at higher engine load, EGR was required to limit the maximum pressure rise rate. From studies of the above scholars, the use of a single n-butanol fuel cannot achieve stable and efficient combustion under all load conditions. Therefore, researchers have proposed to solve the problems related to narrow operating range of n-butanol by blending different active fuels and additives.

Zhou et al.^23^ studied the effect of H_2_O_2_ on the performance of n-butanol HCCI engine at low and medium loads. The results showed that the addition of additive H_2_O_2_ to n-butanol could promote the formation of OH and HO_2_ radicals in low-temperature oxidation reaction stage, and significantly affected the performance of the HCCI engine. When mixture concentration or *T*_*in*_ was low, compared with burning pure n-butanol, the indicated thermal efficiency with the addition of H_2_O_2_ had been greatly improved, and HC and CO emissions were significantly reduced. By adding H_2_O_2_ to n-butanol, operating range of the n-butanol HCCI engine could be widened to low loads. Agbro et al.^24^ proved by simulation analysis that change of OH concentration was the key factor affecting the low-temperature ignition of n-butanol. In PPCI combustion, the influence of fuel auto-ignition quality was much greater than volatility^25^. Zhang et al.^26^ conducted a study on multiple injection PPCI using gasoline/diesel blends. The results showed that when IMEP was between 0.29 MPa and 0.62 MPa, although the blends could reduce average particle size and emission numbers of particulate matter, the early injection of the blends leaded to an earlier combustion phase and NOx emissions were higher than that of the original diesel combustion. Rezaei et al.^27^ found that optimizing parameters such as injection timing and fuel injection ratio could reduce NOx and soot emissions. Studies have shown that the timing of injection has an important impact on CO, NOx and HC emissions. Zhang et al.^28^ also found that under 50% EGR conditions, blends could simultaneously achieve low NOx and soot emissions, but the pressure rise rate and fuel consumption were higher than diesel. At the same time, they found that the blends PPCI could reduce the condensed particulate matter by about 99%, and the blends had obvious advantages in particulate matter emissions until medium load (0.785 MPa BMEP).

At present, there is very little research report on effect of gasoline additive on the combustion and emission characteristics of an n-butanol PPCI engine under different parameters. Therefore, in order to expand operating range of the PPCI engine, taking into account the fuel characteristics and combustion control scale, and promote the practical use of PPCI engines, this paper investigates the combustion and emission characteristics of a certain percentage of gasoline blended with n-butanol PPCI engine under different parameters. In the experiments, the influence of important parameters such as blending ratio, *T*_*in*_ and excess-air coefficient (*λ*) on the combustion and emission characteristics of PPCI were considered.

## Experimental apparatus and procedure

### Experimental setup

In this study, an inline two-cylinder, four-stroke, naturally aspirated, forced water-cooled diesel engine (CT2100Q, China) was modified accordingly, so that the 1st cylinder continues to work in diesel mode, and the 2nd cylinder works in PPCI mode. After adding the developed fuel direct-injection system, the modified engine can conduct the PPCI combustion research. The specifications of test engine are shown in Table 1.

**Table 1:** The specifications of test Engine.

Items	Parameters
Model	CT2100Q
Bore × Stroke	100 mm × 105 mm
Compression ratio	17.0:1
Intake valve opening angle	17°CA BTDC
Intake valve closing angle	43°CA ABDC
Exhaust valve opening angle	47°CA BBDC
Exhaust valve closing angle	17°CA ATDC

**Figure 1:**
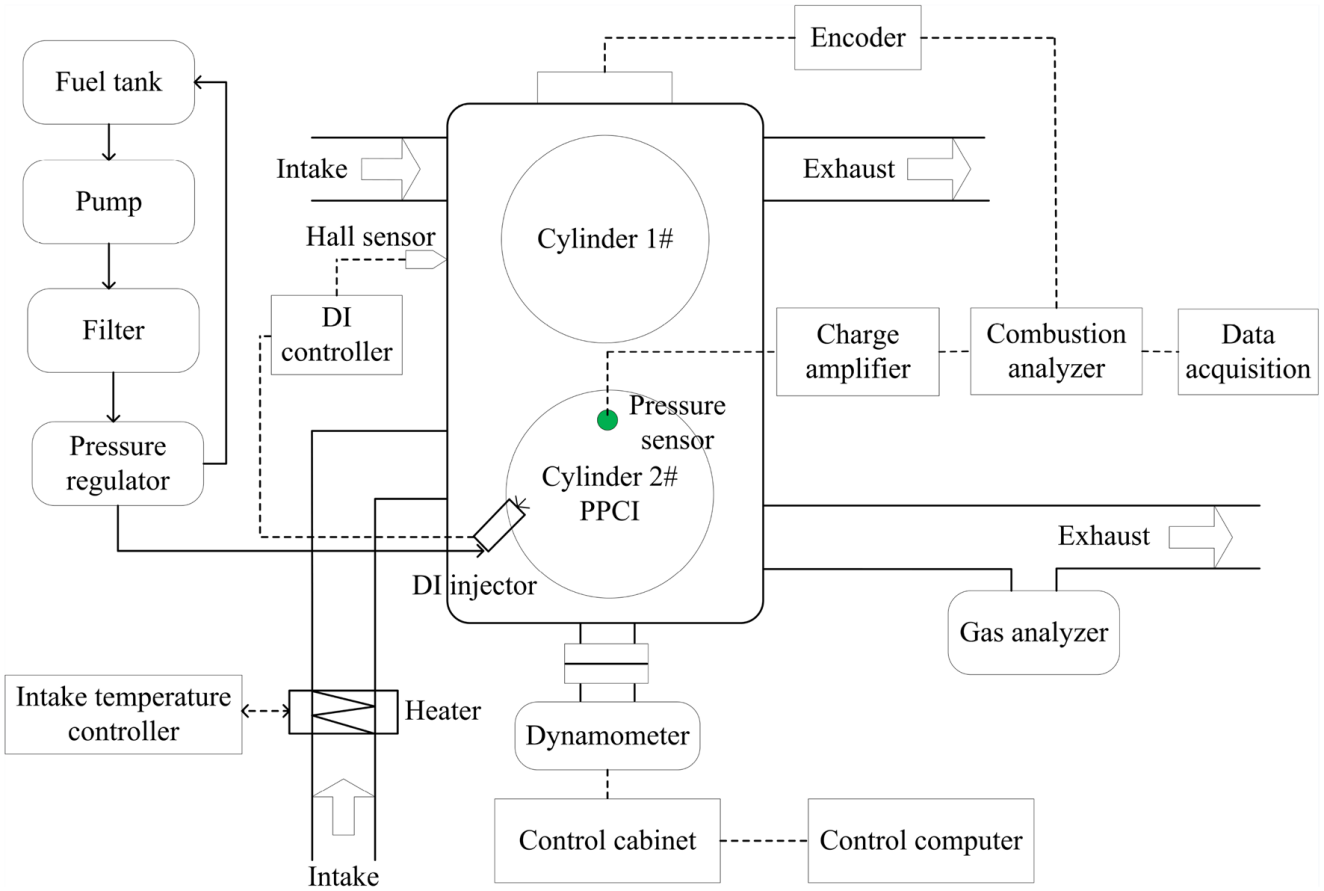
Schematic of engine test apparatus.

The experimental setup is shown in Fig. 1. The intake manifold and exhaust manifold of original diesel engine were removed, the intake and exhaust pipes were installed in the 1st cylinder and in the 2nd cylinder, respectively. So that the two cylinders can work independently, and the collection of emission data is mutually exclusive influence. In addition, n-butanol and gasoline used in this experiment are high-octane fuels, and boiling point of n-butanol is 118 °C. At normal temperature, it cannot form homogeneous mixture with air to achieve PPCI combustion, so the intelligent air intake heating system (KN-2, Wuxi, China) was installed on the intake port of the 2nd cylinder.

The fuel supply system of original 1st cylinder remains unchanged, the fuel supply system of the 2nd cylinder was removed, and one direct injection (DI) fuel set is installed. The DI fuel supply system is mainly composed of a motor, a high-pressure fuel pump, a common-rail pipe, a DI fuel injector and a rail pressure controller. The high-pressure fuel pump is driven by the motor to establish a high-pressure system. The rail pressure is set as 10 MPa.

The test engine was coupled to an eddy current dynamometer (CW25, CAMA, Nanfeng, China). The in-cylinder pressure signal acquisition system is mainly composed of piezoelectric pressure sensor (6052A, Kistler), charge amplifier (5019B, Kistler), AVL combustion analyzer (Kibox 283A, Kistler), photoelectric encoder, decoder, AVL IndiCom V2.8 signal acquisition software and a computer. In tests, the tiny electrical signal collected by the pressure sensor was enhanced by the charge amplifier and then transmitted to the AVL combustion analyzer. At the same time, the photoelectric encoder decoded the output crankshaft position signal and then transmitted it to the AVL combustion analyzer. The combustion analyzer combined the cylinder pressure signal and the crankshaft position signal to generate an in-cylinder pressure variation curve based on the angular domain. The rotation angle resolution of the cylinder pressure acquisition is 0.1°CA. The combustion analyzer is connected to the computer by Ethernet network cable. The host computer acquisition software is applied to monitor the cylinder pressure signal in real time and facilitate the operator to record and save data. A DiGas 4000 exhaust gas analyzer (AVL) is used to detect HC, CO and NOx pollutants.

### Experimental procedure

The fuel injection calibration is performed on the four kinds of test fuels to obtain the relationship between the circulating fuel injection quantity and the fuel injection pulse width. The 1st cylinder original diesel engine is used to preheat the engine and start PPCI combustion mode smoothly, until the temperature of coolant and oil reaches 80 °C and 85 °C, respectively. The steady state tests are repeated more than twice to ensure that the results are within the acceptable experimental uncertainties and repeatable. The details of test procedure and setup can be referred to^29^.

### Test fuels

As an alcohol fuel, n-butanol is a renewable energy and has great development prospects. At the same time, its water absorption rate and corrosivity are lower, and transportation costs are lower. In this paper, four fuels B100, B90G10, B80G20 and B70G30 (B*x*G*y*: volume ratios of n-butanol and gasoline in the blends are *x*% and *y*%, respectively) are used. The n-butanol is produced by Xi'an Tiancheng Chemical Reagent Co., Ltd., the gasoline is 95# gasoline. Their properties are shown in Table 2.

**Table 2:** Properties of n-butanol and gasoline.

Properties	n-butanol	gasoline
Chemical formula	C_4_H_10_O	C_5_ ~ C_18_
Molecular weight	74	99
Research octane number (RON)	95 ~ 100	95
Cetane number (CN)	25	low
Stoichiometric ratio (m/m)	11.2	14.7
Lower heating value (MJ/kg)	33.1	42.5
Heat of vaporization (kJ/kg)	716	297
Density at 20 °C (kg/L)	0.81	0.73
Kinematic viscosity at 20 °C (mm^2^/s)	2.51	0.76
Oxygen content (m%)	22	0
Surface tension at 20 °C(mN/m)	24.6	22.6

### Data processing

In this paper, the combustion phase parameters are defined as *CA10*, *CA50* and *CA90*, which represent the corresponding crank angle when cumulative heat release reached 10%, 50% and 90%. Furthermore, the combustion duration (*CD*) is defined as the period between *CA10* and *CA90*.

In data processing, the in-cylinder pressure data of 100 consecutive working cycles are recorded and averaged. *COV*_*Pmax*_ is used to evaluate engine cyclic variation. In addition, *λ*, heat release rate (*HRR*) and coefficient of variation for peak in-cylinder pressure (*COV*_*Pmax*_) are all calculated by the known formulas. All results related to exhaust emissions (ppm) are converted to g/kWh according to the following equations^33^:

1 $$ {\text{ISNOx }}\left[ {\text{g/kWh}} \right] = {\text{NOx }}\left( {{\text{ppm}}} \right) \cdot \frac{{\left( {m_{air} + m_{fuel} } \right) \cdot 1.587}}{{1000 \cdot Ni\left[ {kW} \right]}} $$

2 $$ {\text{ISCO }}\left[ {\text{g/kWh}} \right] = {\text{CO }}\left( {{\text{ppm}}} \right) \cdot \frac{{\left( {m_{air} + m_{fuel} } \right) \cdot 0.966}}{{1000 \cdot Ni\left[ {kW} \right]}} $$

3 $$ {\text{ISHC }}\left[ {\text{g/kWh}} \right] = {\text{HC }}\left( {{\text{ppm}}} \right) \cdot \frac{{\left( {m_{air} + m_{fuel} } \right) \cdot 0.479}}{{1000 \cdot Ni\left[ {kW} \right]}} $$

where 1.587, 0.966 and 0.479 are respectively the molecular weight of NOx, CO and HC.$$ Ni$$ is the indicated power.

For all these calculations the fuel mass according to the following equations:

4 $$ m_{fuel} = m_{n - butanol} + m_{gasoline} \cdot \frac{{LHV_{gasoline} }}{{LHV_{n - butanol} }} $$

## Results and discussion

### Effect of excess-air coefficient

This test is to study the effect of *λ* on the combustion and emission characteristics of n-butanol/gasoline blends with different blending ratios. During the test, *T*_*in*_ was controlled at 120 °C, *n* was set at 1200 r/min, and the time of fuel direct injection in the cylinder was maintained at 20°CA after intake top dead center. *λ* was 2.0, 2.5 and 3.0, respectively.

#### In-cylinder pressure and HRR

**Figure 2:**
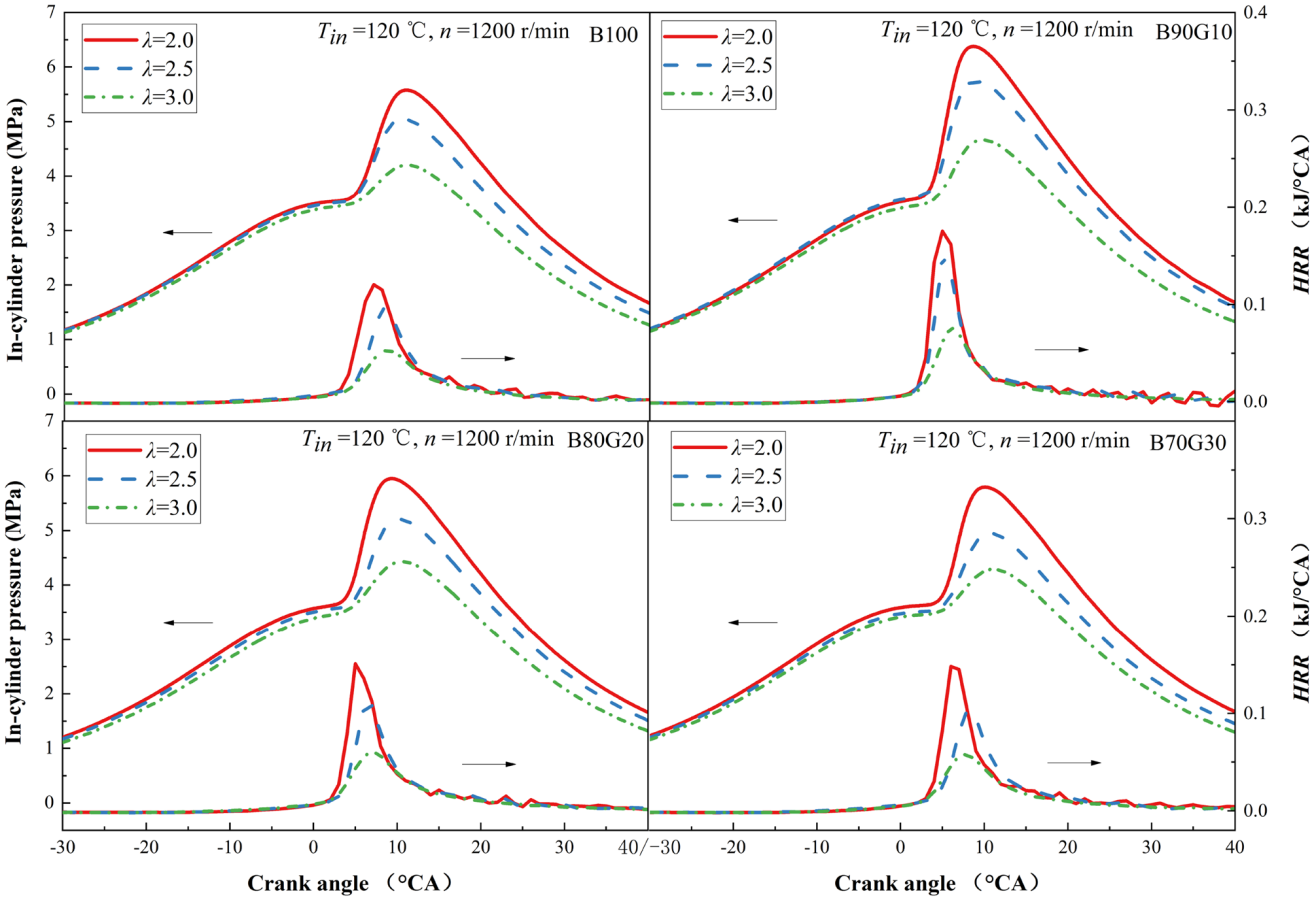
In-cylinder pressure and *HRR* with different *λ*s.

Figure 2 shows the effect of *λ* on the in-cylinder pressure and *HRR* of four test fuels B100, B90G10, B80G20 and B70G30. It can be seen that with the reduction of *λ*, *P*_*max*_ and *HRR*_*max*_ gradually increase. This is because as *λ* decreases, mixture concentration increases, the amount of fuel in the unit volume mixture increases, the chemical reaction rate is accelerated, and the heat release increases, causing the increase of *P*_*max*_ and *HRR*_*max*_. Under the same *λ*, *P*_*max*_ and *HRR*_*max*_ of four fuels increase first and then decrease as gasoline blending ratio increases. The results of pure n-butanol in the experiment are consistent with previous publications^2,23,34^. When *T*_*in*_ is 120 ℃, *λ* is 2.5, and *n* is 1200 r/min, the in-cylinder pressure and *HRR* of B100 are close to the results in references^23^. When the addition ratio of gasoline in the blend is 10%, *P*_*max*_ and *HRR*_*max*_ reach the maximum values. For B90G10, with *λ* changes from 3.0 to 2.0, *P*_*max*_ increases from 4.67 to 6.39 MPa and *HRR*_*max*_ increases from 0.077 kJ/°CA to 0.175 kJ/°CA. It can be seen from the fuel properties that compared with n-butanol, gasoline has a higher calorific value, lower viscosity and latent heat of vaporization. After adding a small proportion of gasoline in n-butanol, atomization quality of the blend is improved and calorific value of the blend is increased. Under the same *T*_*in*_, preparation of combustible mixture is more uniform, which promotes acceleration of the chemical reaction rate, the heat released after fuel oxidation increases, and the in-cylinder pressure rises rapidly, so the *P*_*max*_ and *HRR*_*max*_ increase significantly. The activity of n-butanol is higher than that of gasoline in low-temperature oxidation stage of PPCI. The consumption of n-butanol is mainly affected by OH. At the same time, OH generated during the oxidation of n-butanol promotes the oxidation of gasoline^30^. When gasoline blending ratio is greater than 10%, the evaporation and atomization effects of n-butanol/gasoline blends are better, and heating value of the blends increases, while the content of n-butanol in the blends decreases at this time, and the reduction in the amount of OH generated in low-temperature reaction stage is not conducive to oxidation of the blends, so that the combustion reaction rate decreases and *HRR* slows down, which causes *P*_*max*_ and *HRR*_*max*_ to decrease, and at the same time, the corresponding crankshaft angle positions move backward. As can be seen that lifting point of *HRR* curve of four fuels at different *λ*s are basically the same, and the three blends are not much different from B100. However, under three different *λ*s, the *P*_*max*_ and *HRR*_*max*_ of PPCI combustion fueled with n-butanol/gasoline are greater than those of B100, especially B90G10. A certain proportion of gasoline added to n-butanol can improve the combustion of n-butanol, but as gasoline blending ratio increases, the in-cylinder pressure and *HRR* tend to decrease.

#### In-cylinder temperature

**Figure 3:**
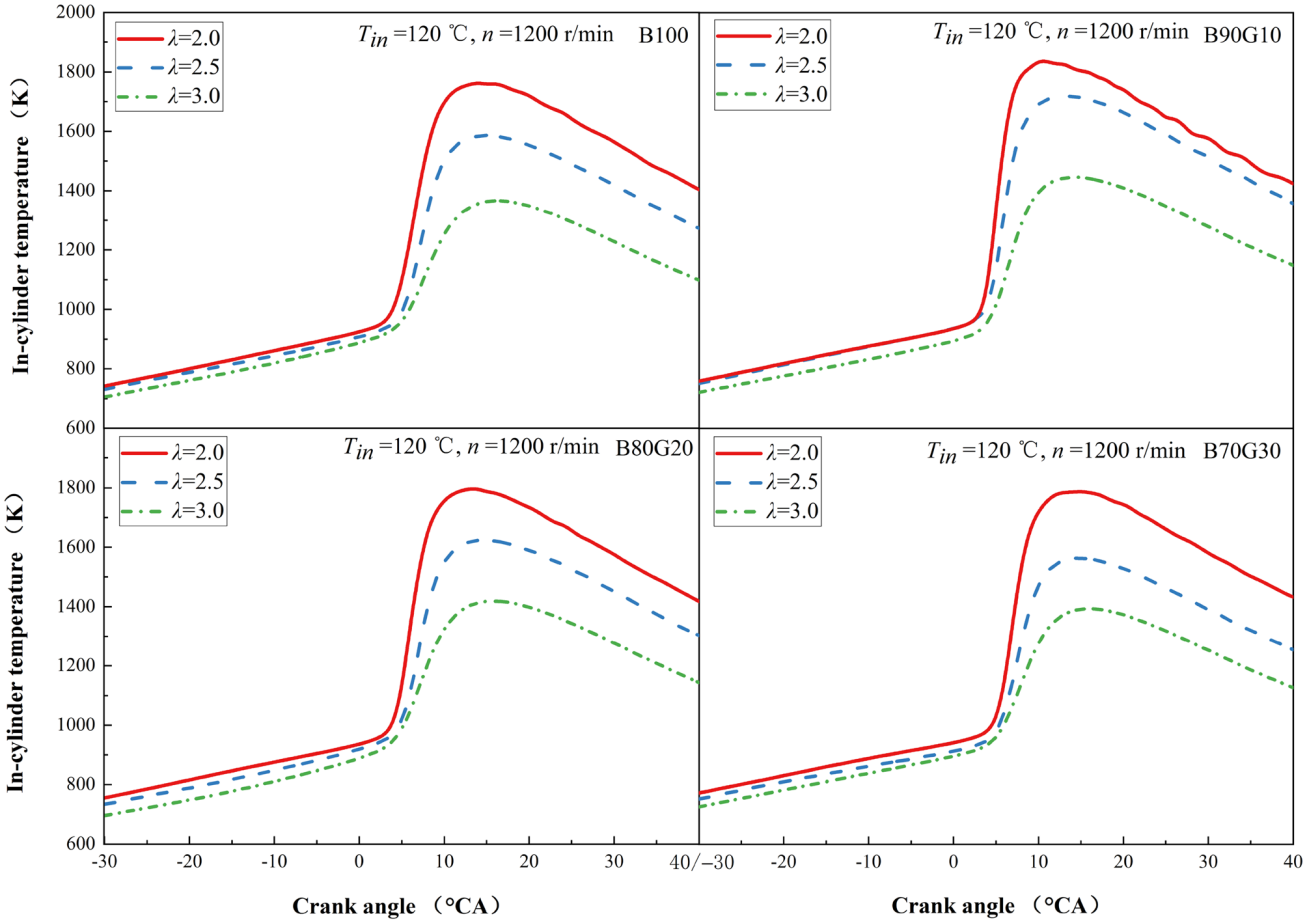
In-cylinder temperature with different *λ*s.

Figure 3 shows the influence of *λ* on the in-cylinder temperature of four test fuels. It can be seen that with the reduction of *λ*, the *T*_*max*_ of four fuels increases significantly, the crank angle position corresponding to *T*_*max*_ moves slightly forward, and the in-cylinder temperature curve gradually becomes steeper. Under the same mixture concentration, with the increase of gasoline blending ratio, the *T*_*max*_ of four fuels increases first and then decreases. When the addition ratio of gasoline is 10%, *T*_*max*_ increases by about 390 K with *λ* changes from 3.0 to 2.0, and *T*_*max*_ reaches the maximum value. This is mainly because the concentration increases as *λ* decreases, the amount of fuel in the unit volume increases, and the total heat release from combustion is bound to increase, causing *T*_*max*_ to increase. At the same time, the reaction preparation time is shortened, the cycle heat release is significantly increased, and the in-cylinder temperature rises rapidly, making the position of crank angle at which *T*_*max*_ is slightly advanced. In addition, at different *λ*s, the sharp rise inflection point of B90G10 in-cylinder temperature is earlier than the other three fuels to a certain extent. When *λ* is 2.0, 2.5, and 3.0, the inflection point of B90G10 corresponds to crank angle earlier than that of B100 by 1.4°CA, 2.5°CA and 0.3°CA. This is because after adding gasoline to n-butanol, the blend calorific value is increased, atomization quality is improved, preparation of combustible mixture is more uniform, combustion is more complete, heat release increases and concentrates, *HRR*_*max*_ increases, at last in-cylinder temperature rises accordingly. When addition ratio of gasoline is greater than 10%, the proportion of n-butanol in the blends gradually decreases, and the blends overall activity decreases, which is not conducive to generation and accumulation of active groups in low-temperature oxidation reaction stage, resulting in the blend heat release in high-temperature reaction stage is reduced, and in-cylinder temperature is also reduced.

#### Combustion phase

**Figure 4:**
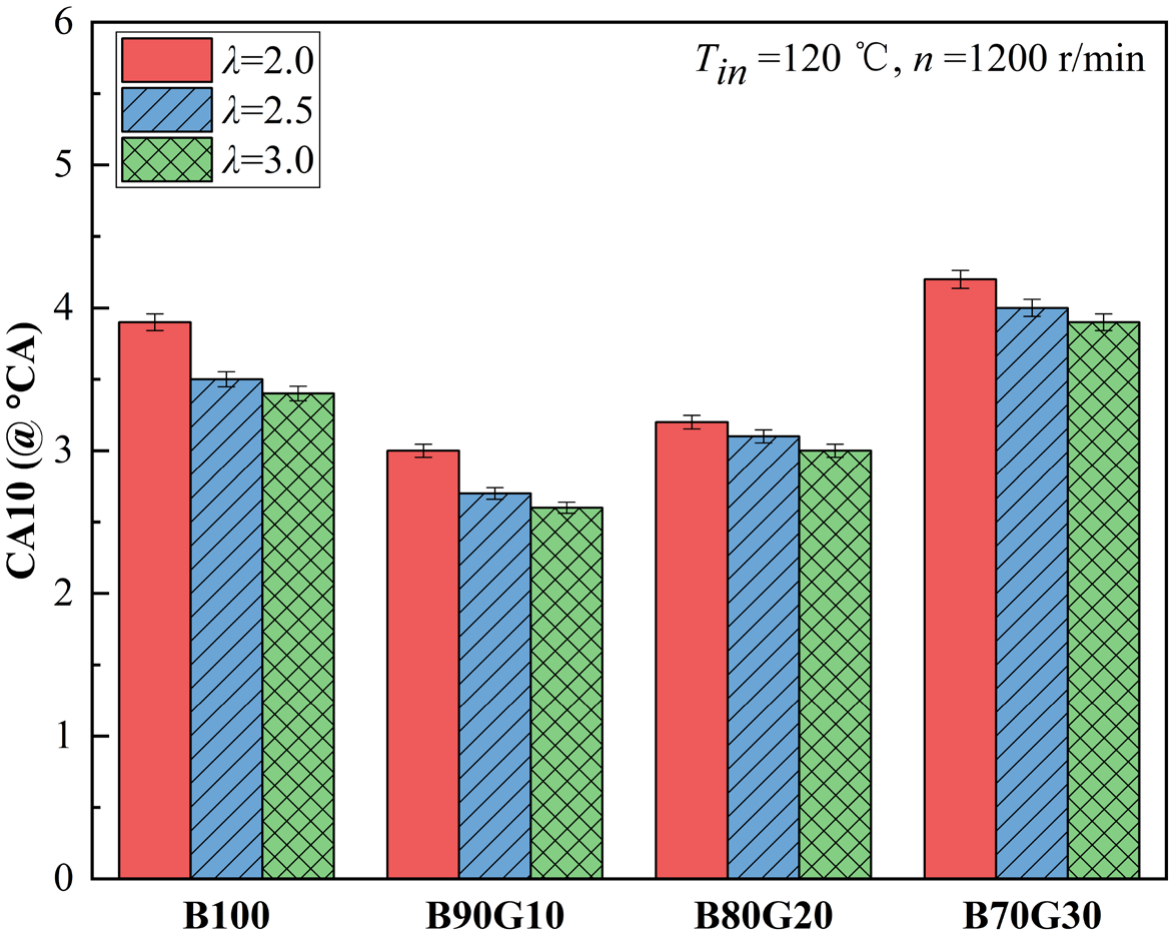
*CA10* with different *λ*s.

Figure 4 shows the effect of *λ* on *CA10* of four test fuels. It can be seen that with the increase of mixture concentration, *CA10* of four fuels lags only slightly, and basically does not change. The reasons are as the following: on the one hand, the concentration of mixture increases, the amount in the unit volume mixture increases, and the proportion of molecules participating in reaction increases, exacerbating the collision between molecules, the chemical reaction rate is accelerated, which has the effect of promoting ignition; on the other hand, at this intake temperature, the mixture concentration increases, the amount of heat absorbed by vaporization increases, the in-cylinder temperature decreases, the atomization quality becomes poor, and the prepared mixture is not uniform enough, which is not conducive to ignition. Under the combined effect influence, mixture concentration may eventually increase and *CA10* is slightly delayed. It also can be seen that under the same *λ*, as gasoline blending ratio increases, *CA10* of four fuels shows a gradual lagging trend after the move forward at first. When gasoline addition ratio is 10%, the forward range of *CA10* is the largest, but the forward range gradually decreases as mixture concentration becomes leaner. *CA10* of four fuels is after top dead center. This is because *CA10* has a strong dependence on in-cylinder thermodynamic state at the end of compression stroke. After adding gasoline to n-butanol, the blend latent heat of vaporization is reduced, atomization quality is improved, and preparation of combustible mixture is more uniform, which is beneficial to the generation and accumulation of active groups in the blend during low-temperature oxidation reaction phase. It accelerates chemical reaction rate, in-cylinder temperature is higher at the end of compression, and the ignition time is advanced. As gasoline proportion continues to increase, the blends cetane number gradually decreases, mixture reactivity decreases significantly, ignition is difficult, and *CA10* gradually lags. It can be seen that under three different *λ*s, adding a small proportion of gasoline to n-butanol can change the combustion phase, making *CA10* advance. However, adding gasoline over a certain proportion may delay *CA10*.

**Figure 5:**
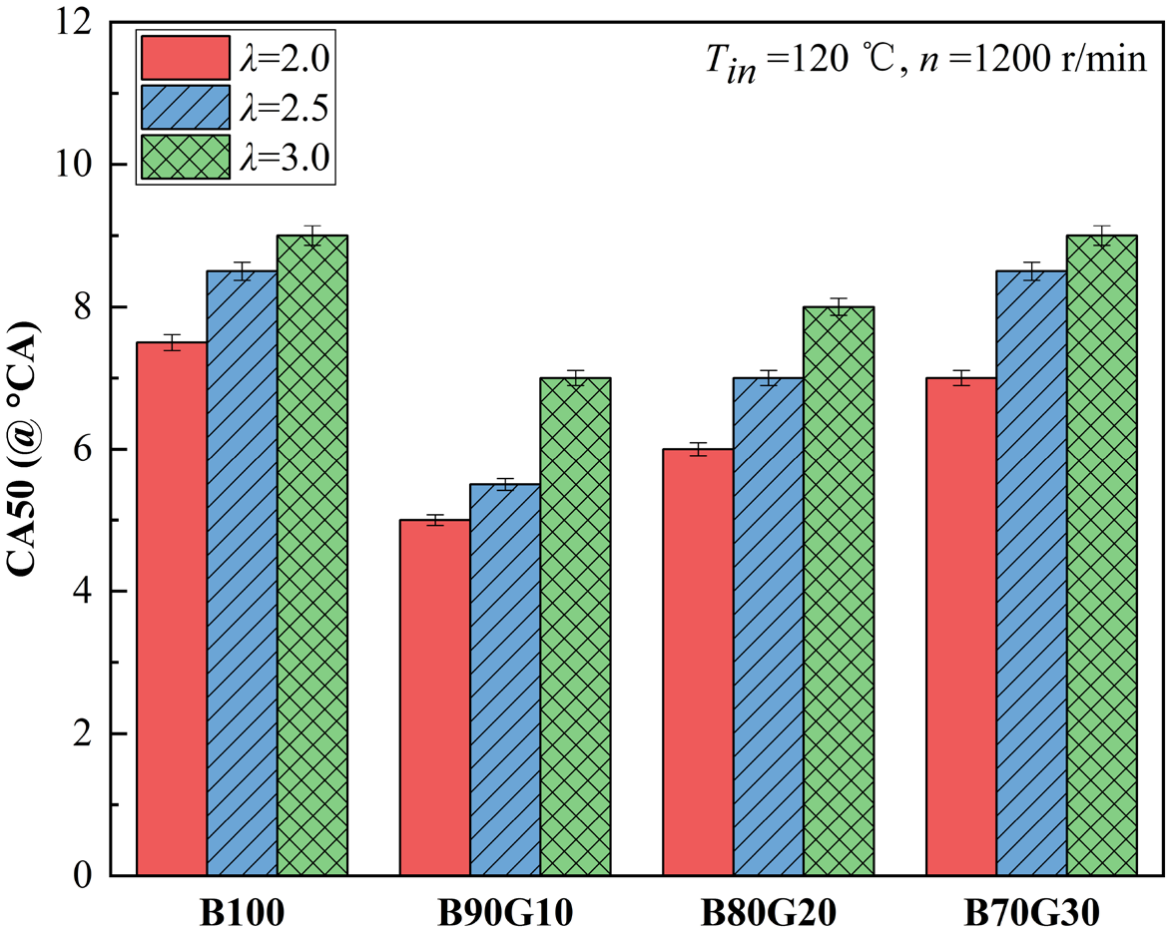
CA50 with different *λ*s.

**Figure 6:**
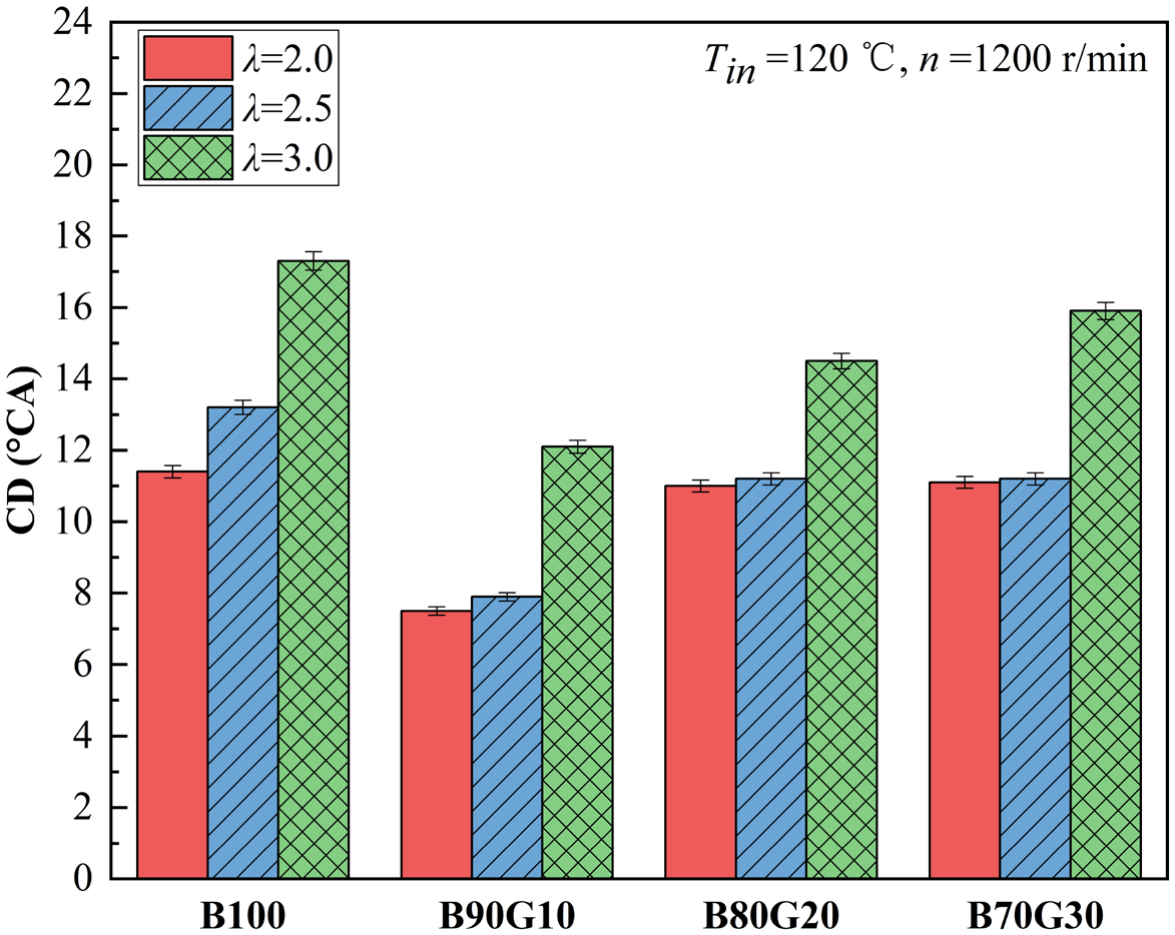
*CD* with different *λ*s.

Figures 5 and 6 show the effect of *λ* on *CA50* and *CD* of four test fuels, respectively. It can be seen that as *λ* increases, *CA50* of four fuels gradually lags, and *CD* gradually extends. This is because with the increase of *λ*, mixture concentration becomes leaner, number of activated molecules decreases, chemical reaction rate decreases, combustion exotherm slows down and gradually worsens, and *CA50* lags gradually, and *CD* gradually extends. It can be seen that under the same *λ*, with the increase of gasoline blending ratio, change of four fuels *CA50* is basically similar to *CA10*, showing a gradual lagging trend after move forward at first. When gasoline proportion is 10%, *CA50* advances the most. After adding a small proportion of gasoline in n-butanol, *CA10* of the blends is advanced, the combustion heat release time is advanced, and in-cylinder reaction rate of each element is accelerated, causing *CA50* to move forward accordingly. When gasoline proportion in the blend is greater than 10%, the blends activity decreases, *CA10* gradually lags, and the corresponding *CA50* also gradually moves backward. It also can be seen that under different *λ*s, as gasoline proportion increases, *CD* of four fuels shows a trend of decreasing first and then increasing. *CD*s of B90G10, B80G20 and B70G30 are shorter than B100, and *CD* of B90G10 is the shortest. When *λ* is 2.0, 2.5 and 3.0, *CD* of B90G10 is 7.5°CA, 7.9°CA and 12.1°CA. This is because the addition of a small proportion gasoline in n-butanol promotes the formation of homogeneous mixture. In low-temperature oxidation reaction stage, it helps n-butanol to generate more OH, which accelerates fuel decomposition and heat release, resulting in *CD* shorter. When the addition ratio of gasoline in the blends is greater than 10%, although the mixture prepared is more uniform, the content of n-butanol in the blends is reduced, the activity of the blends is reduced, and number of OH generated in low-temperature reaction stage is reduced, which suppresses fuel oxidative decomposition, combustion reaction rate gradually decreases, resulting in *CD* prolonged. It can be seen that adding a small proportion of gasoline to n-butanol can change the combustion phase, resulting in *CA50* advance and *CD* shorten. However, adding gasoline over a certain proportion may delay *CA50* and extend *CD*. Overall, compared with B100, *CA50* of three blends are advanced and *CD* of three blends are shortened under the same *λ*.

#### Cyclic variation

**Figure 7:**
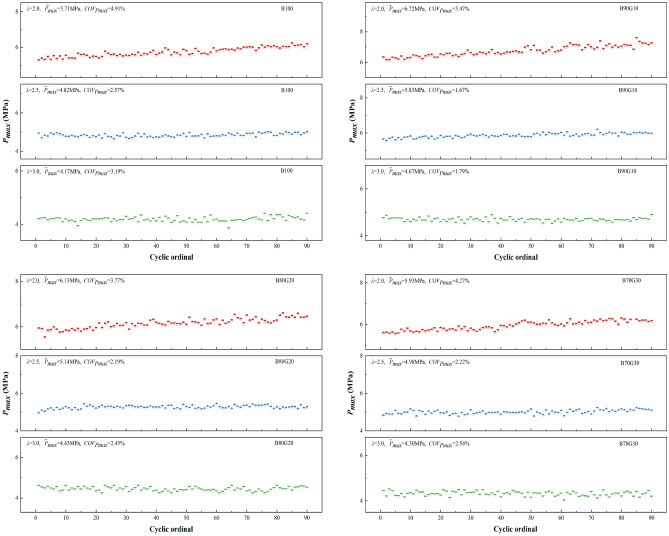
Cyclic variation with different *λ*s.

Figure 7 shows the effect of *λ* on $\overline{P}_{max}$ and *COV*_*Pmax*_ of four test fuels. It can be seen that as mixture concentration increases, $\overline{P}_{max}$ of four blends monotonously increases, and *COV*_*Pmax*_ shows a trend of large on both sides and small in the middle. This is because when* λ* increases, mixture concentration decreases, and the amount of heat release decreases, resulting in unstable combustion and larger *COV*_*Pmax*_. When *λ* is low, mixture concentration is high, and mixture is knocked in the combustion, which deteriorates the combustion, reduces the stability of PPCI engine operation, and increases its cycle variation. Therefore, within a certain range, intake temperature and proper mixture concentration are critical to its smooth operation. It also can be seen that under the same *λ*, with the increase of gasoline blending ratio, $\overline{P}_{max}$ of four blends first increases and then decreases, while the change trend of *COV*_*Pmax*_ is the opposite. When gasoline addition ratio is 10%, $\overline{P}_{max}$ is the highest and *COV*_*Pmax*_ is the lowest. When *λ* is 2.0, 2.5 and 3.0, $\overline{P}_{max}$ of B90G10 is increased by 17.7%, 21.0% and 12.0% compared to B100, and *COV*_*Pmax*_ is as low as 3.47%, 1.67% and 1.79%, respectively. This is because of a small proportion gasoline addition in n-butanol, preparation of combustible mixture is more uniform, fuel reaction is more complete, heat release is increased and concentrated, in-cylinder temperature increases rapidly, and the continuous high-temperature environment can be maintained during the engine working cycle, so that combustion operation of the PPCI engine is stable. When added gasoline amount continues to increase, number of active groups accumulated in low-temperature stage decreases, and heat release amount decreases, resulting in lower combustion reaction rate, a reduction in the temperature and pressure, and lower combustion stability. So the range of cyclic variation is increased. It can be seen that under the same *λ*, *COV*_*Pmax*_ of three blends are all smaller than that of B100, especially B90G10. The combustion stability of blends is better. So, adding a small proportion of gasoline to n-butanol can improve its combustion stability.

#### Emission characteristics

**Figure 8:**
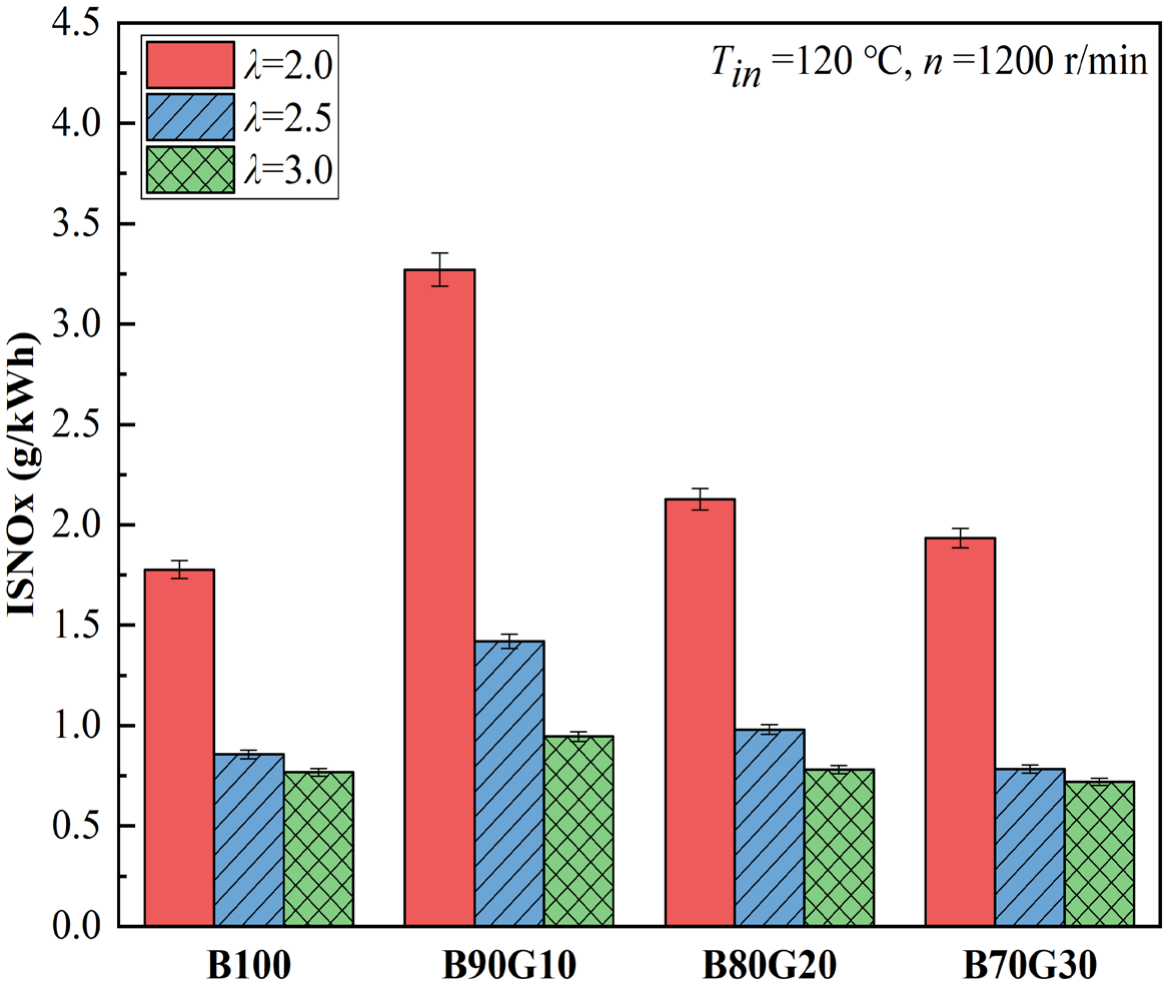
NOx emissions with different *λ*s.

Figure 8 shows the effect of *λ* on NOx emissions of four test fuels. It can be seen that as *λ* decreases, NOx emissions increase. This is because the mixture concentration increases with the reduction of *λ*, and in-cylinder temperature gradually increases, which is beneficial to NOx formation. It also can be seen that NOx emissions tend to increase slightly first and then gradually decrease with the increase of gasoline blending ratio. In general, fuel blending ratio has little effect on NOx emissions, and NOx emissions of each blending ratio fuel all are low and close to zero. When *λ* is 2.0, NOx emission of B90G10 is the largest. NOx formation is closely related to the in-cylinder temperature, oxygen concentration, and high-temperature reaction residence time. The PPCI engine uses partially homogeneous mixture. Although its oxygen is sufficient during combustion process, the in-cylinder combustion temperature is low and the distribution is even. The high-temperature condition required for NOx generation is avoided. At the same time, when the PPCI engine is combusted, the combustible mixture is almost simultaneously compressed and ignited. Its combustion speed is extremely fast and *CD* is short, which shorten the time on fuel stays in high temperature, thereby suppressing the generation of NOx. According to the previous analysis, compared with B100, *HRR*_*max*_ of n-butanol/gasoline blends are larger, and in-cylinder temperature are relatively higher, so NOx emission increases. The *T*_*max*_ of B90G10 reaches 1868 K, which is higher than the critical temperature for NOx generation of 1800 K, so NOx emission is increased, but at this time the combustion reaction rate is faster, and the residence time of high-temperature reaction is shorter. Therefore, increase in NOx emission is limited. It can be seen that under the same *λ*, NOx emissions of three blends are all higher than B100, especially B90G10.

**Figure 9:**
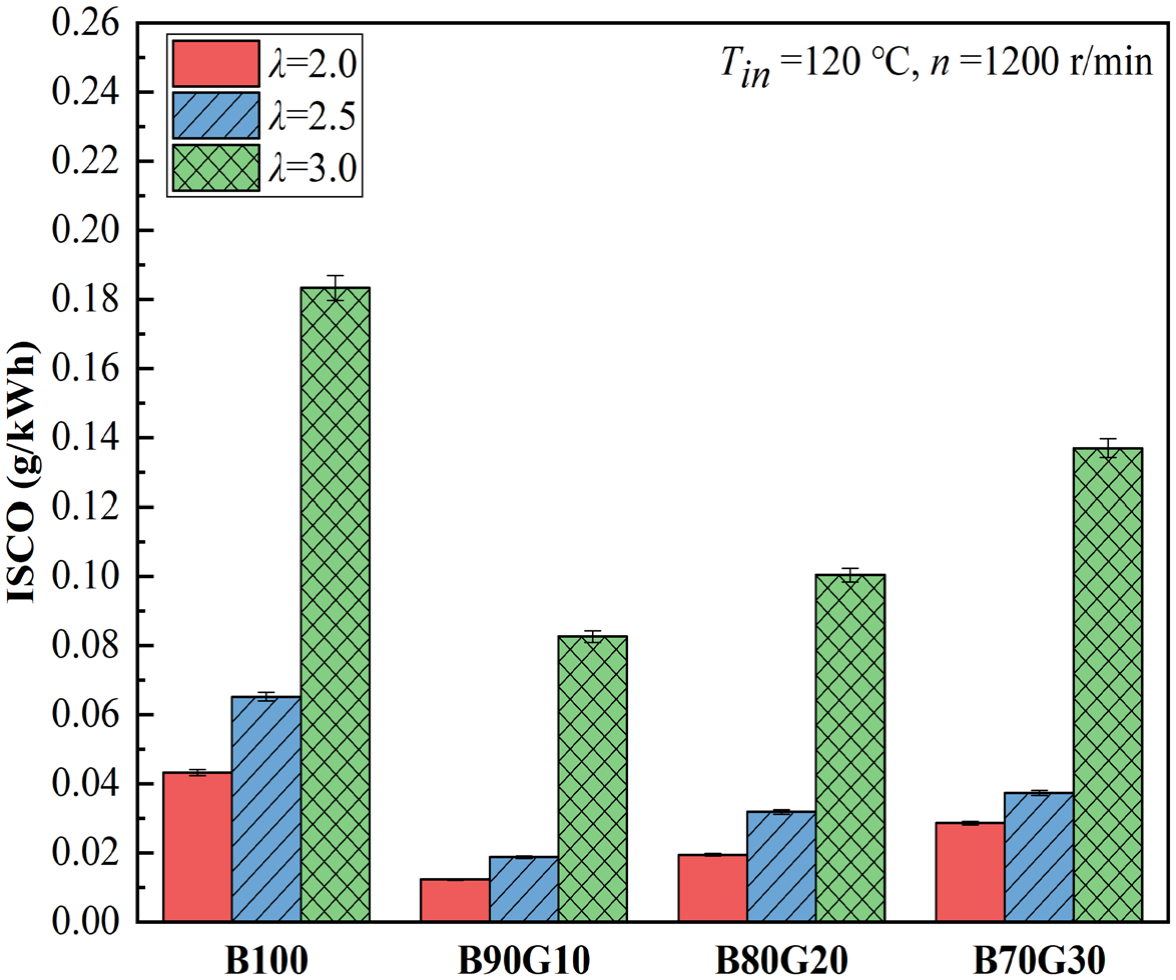
CO emissions with different *λ*s.

**Figure 10:**
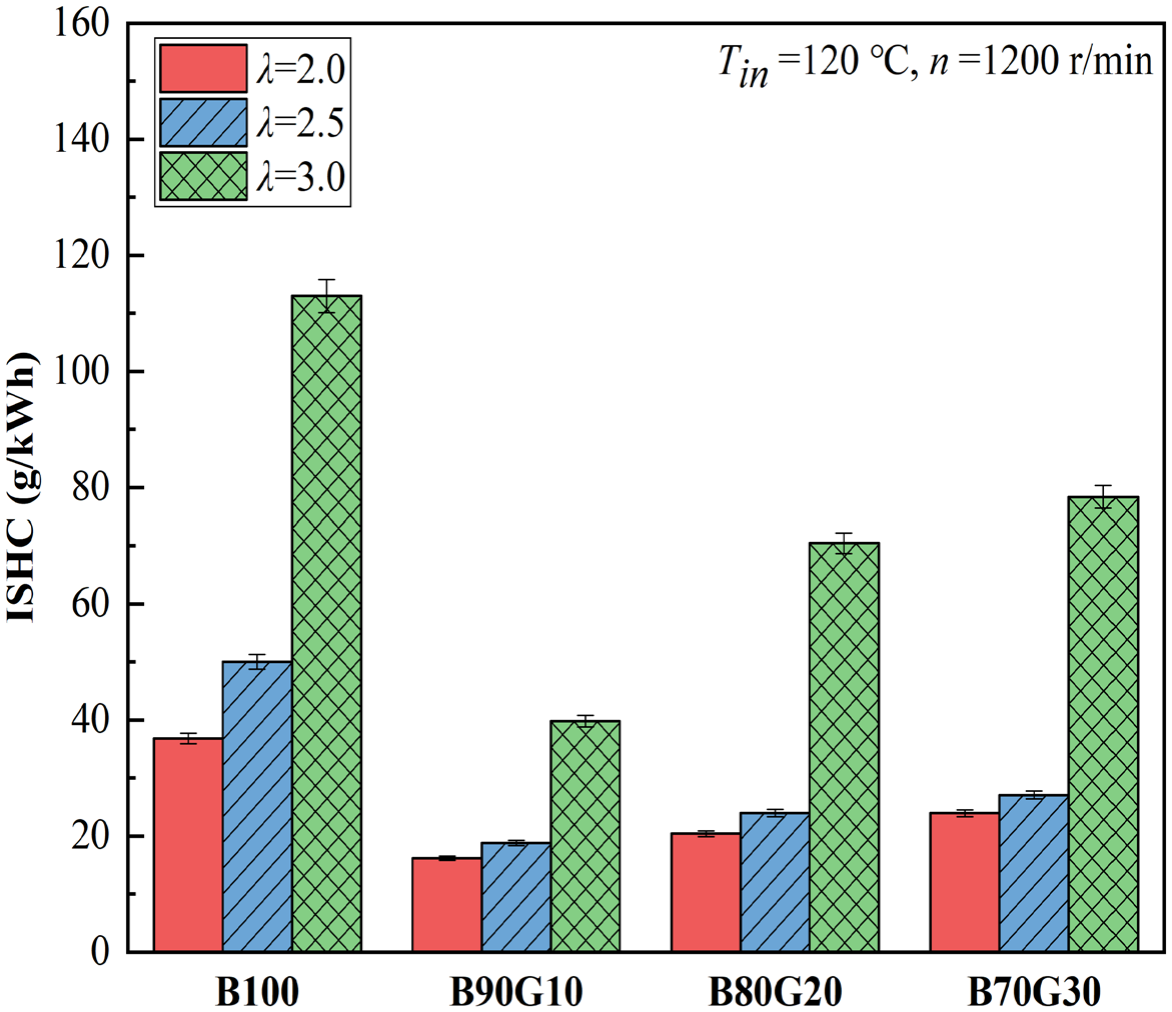
HC emissions with different *λ*s.

Figures 9 and 10 show the effect of *λ* on HC and CO emissions of four test fuels, respectively. It can be seen that with the reduction of *λ*, HC and CO emissions of four test fuels are gradually reduced. As one of the intermediate products of the combustion process, CO is the result of incomplete oxidation. Under homogeneous and lean mixing condition, its generation is closely related to in-cylinder temperature. It can be seen that as blending ratio of gasoline increases, CO emissions decrease first and then gradually increase. When gasoline proportion is 10%, CO emission is the lowest at each mixture concentration. This is because after adding a small proportion gasoline, the prepared mixture is more uniform, and in-cylinder combustion temperature is higher at the end of compression, which creates a favorable environment for CO oxidation. However, when gasoline addition ratio in the blends is greater than 10%, activity of the blends is reduced and ignition is difficult. A large amount of unburned mixture enters the gaps and cannot be fully burned. At the same time, in-cylinder temperature drops, which hinders the progress of CO oxidation reaction, so CO_2_ cannot be produced, resulting in a gradual increase of CO. It also can be seen that as gasoline blending ratio increases, HC emissions show a similar change trend as CO. The generation factors of HC mainly include cylinder-wall chilling effect and narrow-gap effect. When gasoline proportion is 10%, HC emission is the lowest at each mixture concentration. From the previous analysis, it can be seen that after adding a small proportion gasoline in n-butanol, in-cylinder pressure and temperature are increased, so that cylinder-wall chilling effect and narrow-gap effect are weakened, and HC emissions are reduced. It can be seen that under the same *λ*, CO and HC emissions of three blends are lower than B100, especially B90G10. Obviously, the combustion of n-butanol/gasoline blends may reduce CO and HC emissions. This is the opposite of the result of NOx emissions.

### Effect of intake temperature

This test mainly studies the influence of *T*_*in*_ on the combustion process of PPCI engine with n-butanol/gasoline blends at different blending ratios. The test used intake air heater to control *T*_*in*_ at 110 °C, 120 °C, 130 °C and 140 °C, *n* was set at 1200 r/min, *λ* was 2.5, and fuel direct injection time was at 20°CA after intake top dead center.

#### In-cylinder pressure and HRR

**Figure 11:**
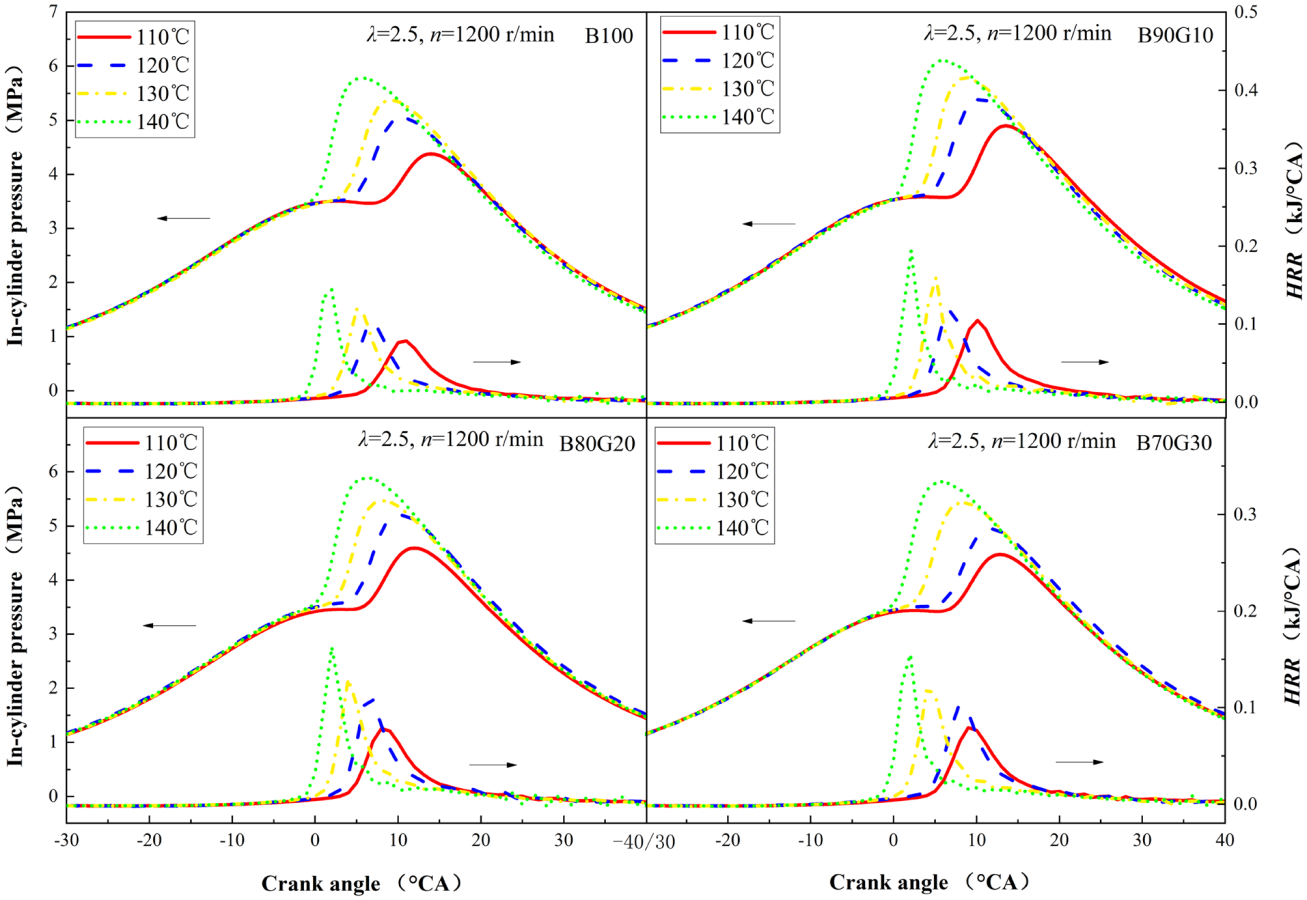
In-cylinder pressure and *HRR* with different *T*_*in*_s.

Figure 11 shows the influence of *T*_*in*_ on in-cylinder pressure and *HRR* of four test fuels. It can be clearly seen that with the increase of *T*_*in*_, the *P*_*max*_ and *HRR*_*max*_ gradually increase, and crank angle position corresponding move forward, heat release is more concentrated. And when *T*_*in*_ is 140 ℃, *λ* is 2.5, and *n* is 1200 r/min, the in-cylinder pressure and *HRR* of B100 are close to the results in references^34^. It can be seen that n-butanol combustion is greatly affected by intake temperature. Under normal intake temperature and pressure conditions, pure n-butanol is difficult to auto-ignite. Due to the influence of n-butanol reactivity and OH groups, only a small part of n-butanol participates in low-temperature branching, which leads to increased ignition delay time at low temperatures^31,32^. For B90G10, when *T*_*in*_ is increased from 110 °C to 140 °C, the *P*_*max*_ is increased from 4.91 MPa to 6.12 MPa. The corresponding crank angle is moved forward by 7°CA. The *HRR*_*max*_ is increased from 0.105 kJ/°CA to 0.194 kJ/°CA, and the corresponding crank angle is advanced by 8°CA. The above phenomenon occurs because with the increase of *T*_*in*_, fuel evaporation and atomization are promoted, and the prepared mixture is more uniform. On the other hand, the increase of *T*_*in*_ increases the proportion of activated molecules in the mixture. The internal energy of the activated molecules increases, which intensifies the collision between molecules, thereby speeding up chemical reaction rate and shortening combustion reaction time, so that the heat release increases, the in-cylinder pressure increases rapidly, and the *P*_*max*_ and *HRR*_*max*_ increase. As can be seen that under the same *T*_*in*_, the *HRR*_*max*_ of PPCI combustion fueled with n-butanol/gasoline blends are greater than B100, especially B90G10. A certain proportion of gasoline added to n-butanol can improve the combustion characteristics of n-butanol, but as gasoline blending ratio increases, the *P*_*max*_ and *HRR*_*max*_ tend to decrease.

It can be seen from the previous analysis that a small proportion gasoline added to n-butanol improves the atomization quality and increases fuel heating value. Under the same *λ*, compared to B100, preparation of combustible mixture is more uniform, which promotes the acceleration of chemical reaction rate, increases heat released after fuel oxidation, and in-cylinder pressure rises rapidly, so the *P*_*max*_ and *HRR*_*max*_ significantly increase. When gasoline blending ratio is greater than 10%, the content of n-butanol decreases, and OH amount generated in low-temperature reaction stage decreases, which is not conducive to fuel-oxidation. It causes combustion reaction rate to decrease and heat release rate to slow down, resulting in *P*_*max*_ and *HRR*_*max*_ drop, and at the same time the corresponding crankshaft angle position moves backward.

#### In-cylinder temperature

**Figure 12:**
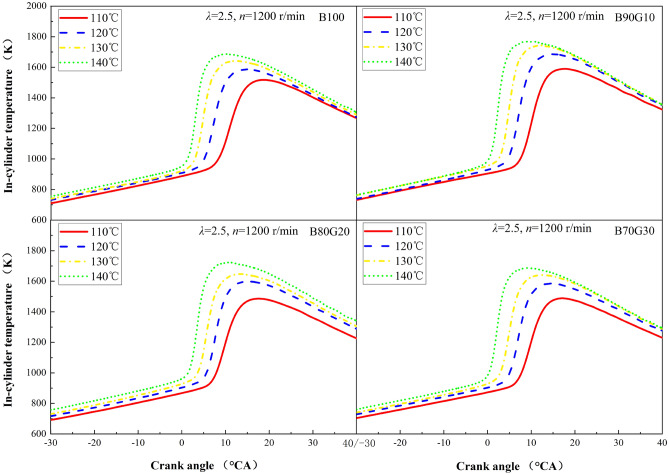
In-cylinder temperature with different *T*_*in*_s.

Figure 12 shows the influence of *T*_*in*_ on in-cylinder temperature of four test fuels. It can be seen that the *T*_*max*_ gradually increases with the increase of *T*_*in*_, and the corresponding crank angle also continuously moves forward. For B90G10, as *T*_*in*_ increases from 110 °C to 140 °C, *T*_*max*_ is increased by 178 °C, and the corresponding crank angle is advanced by 9°CA. This is because with the increase of *T*_*in*_, number of activated molecules and internal energy of the blends increase, which not only facilitates the formation of homogeneous mixture, but also accelerates the reaction rate. The combustion time is shortened, heat released amount is increased and concentrated, so that in-cylinder temperature rises and the *T*_*max*_ time appears moves forward. It can be seen from the previous analysis that after adding gasoline to n-butanol, the atomization quality is improved, combustible mixture preparation is more uniform, and the combustion is more complete. At the same time, *HRR*_*max*_ increases, at last in-cylinder temperature rises accordingly. When gasoline addition ratio is greater than 10%, the overall activity of blends decreases, which is not conducive to the generation and accumulation of active groups in low-temperature oxidation reaction stage. Therefore, in-cylinder temperature is reduced.

#### Combustion phase

**Figure 13:**
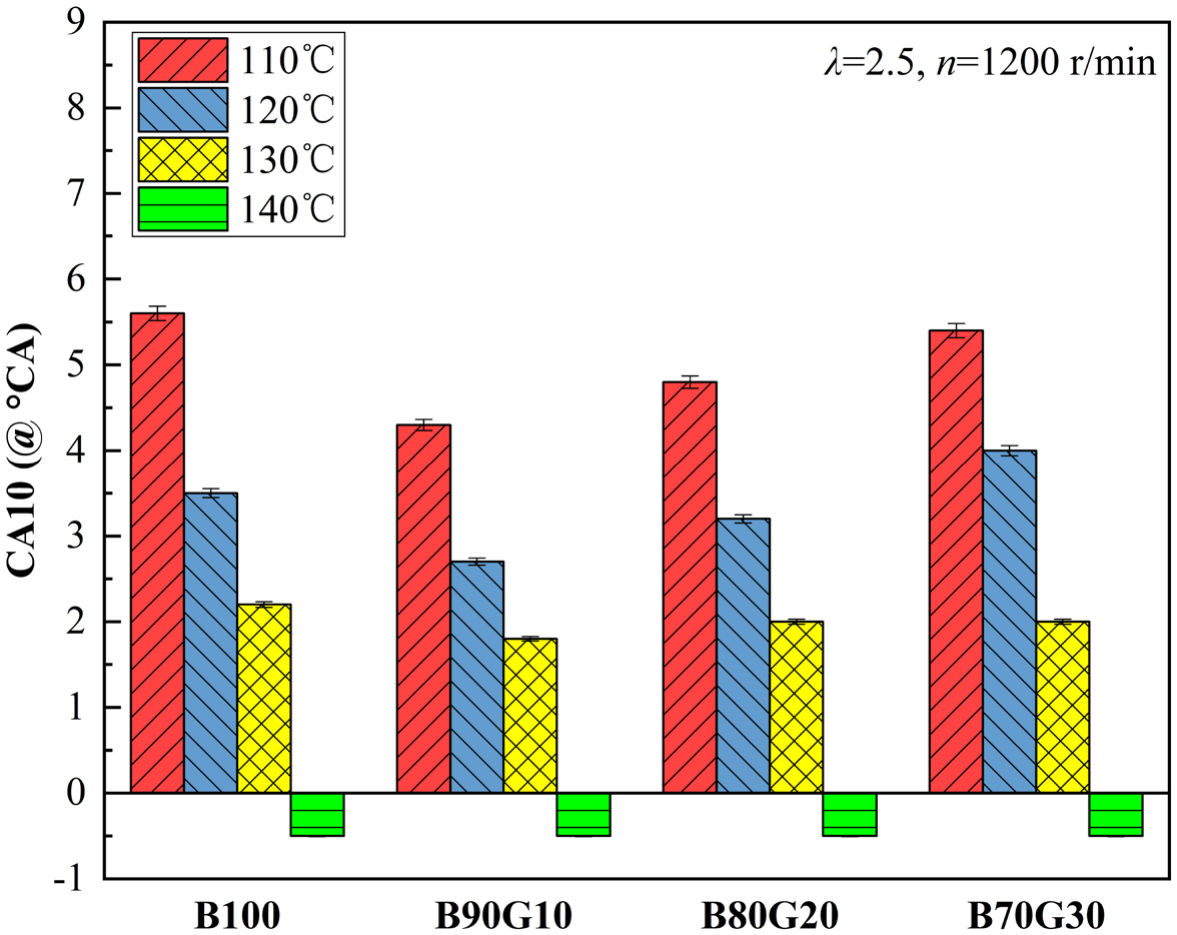
*CA10* with different *T*_*in*_s.

Figure 13 shows the influence of *T*_*in*_ on *CA10* of four test fuels. It can be seen that with the increase of *T*_*in*_, *CA10* of four fuels are advanced. When *T*_*in*_ is 140 °C, *CA10* are all before top dead center. For B90G10, when *T*_*in*_ is 110 °C, *CA10* is at about 4.3°CA. When *T*_*in*_ rises to 130 °C, *CA10* reaches at about 1.8°CA near top dead center. When *T*_*in*_ continues to rise to 140 °C, *CA10* is at about 0.5° CA before top dead center. This is because with the increase of *T*_*in*_, the number of activated molecules and internal energy increase, which makes the mixture easier ignition naturally, the reaction exothermic time advances. *CA10* has a strong dependence on in-cylinder thermodynamic state at the end of compression stroke. After adding gasoline to n-butanol, the atomization quality is improved, and the prepared combustible mixture is more uniform, which is beneficial to the generation and accumulation of active groups in low-temperature oxidation reaction stage. At the end of compression, in-cylinder temperature is higher and ignition time is advanced. As the proportion of added gasoline continues to increase, cetane number of the blends gradually decreases, the reactivity of mixture decreases significantly and *CA10* gradually lags behind.

**Figure 14:**
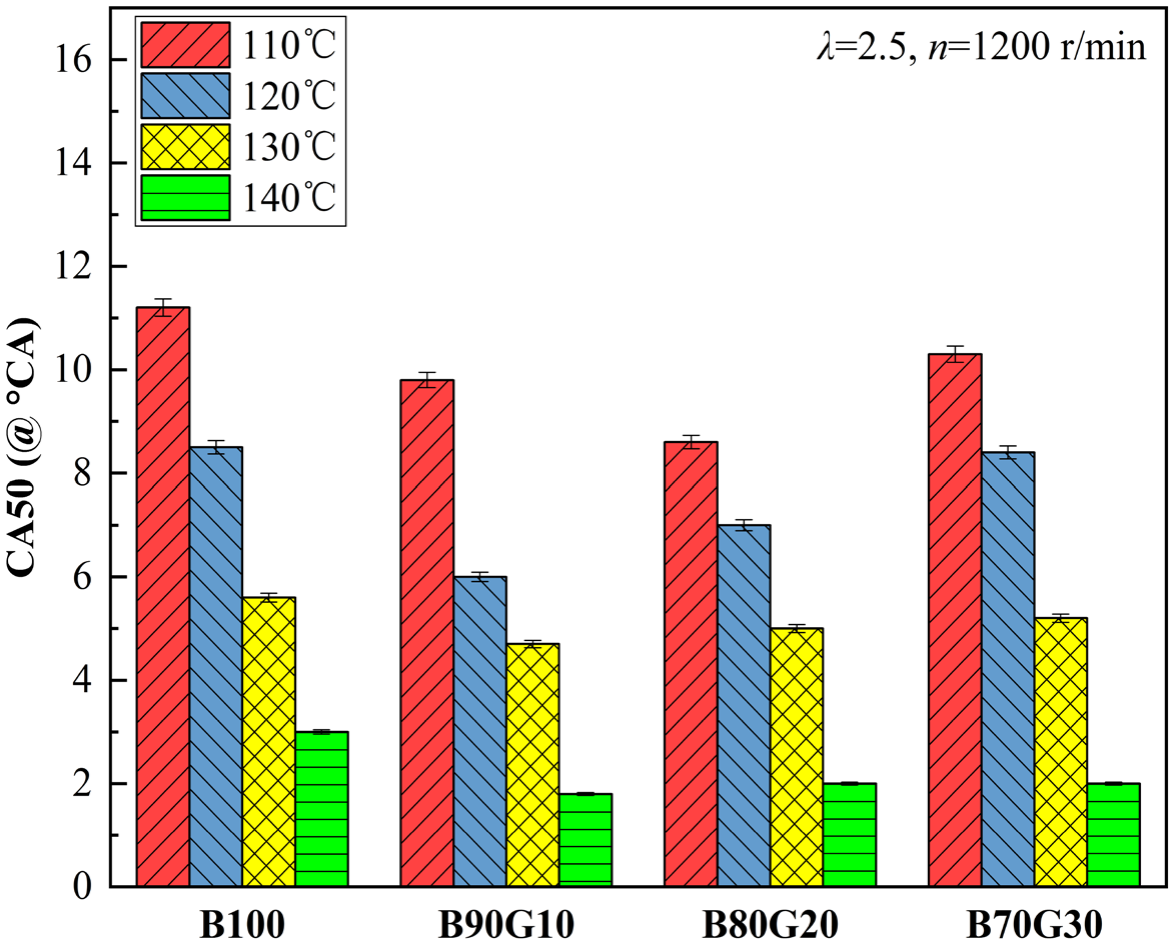
*CA50* with different *T*_*in*_s.

Figure 14 shows the effect of *T*_*in*_ on *CA50* of four test fuels. It can be seen that the change trend of *CA50* is similar to that of *CA10*. With the increase of *T*_*in*_, *CA50* gradually moves forward. For B90G10, *T*_*in*_ is increased from 110 °C to 140 °C, and *CA50* is advanced by about 8°CA. This is because with the increase of *T*_*in*_, the combustion reaction rate is accelerated, the time required to burn 50% mixture is significantly shortened, and *CA50* moves forward. After adding a small proportion of gasoline to n-butanol, *CA10* of blends advances, time of combustion exotherm advances, and reaction rate of each in-cylinder elementary element accelerates, making *CA50* move forward accordingly. When gasoline addition ratio is greater than 10%, the activity of blends decreases, *CA10* gradually lags behind, and the corresponding *CA50* gradually moves backward.

**Figure 15:**
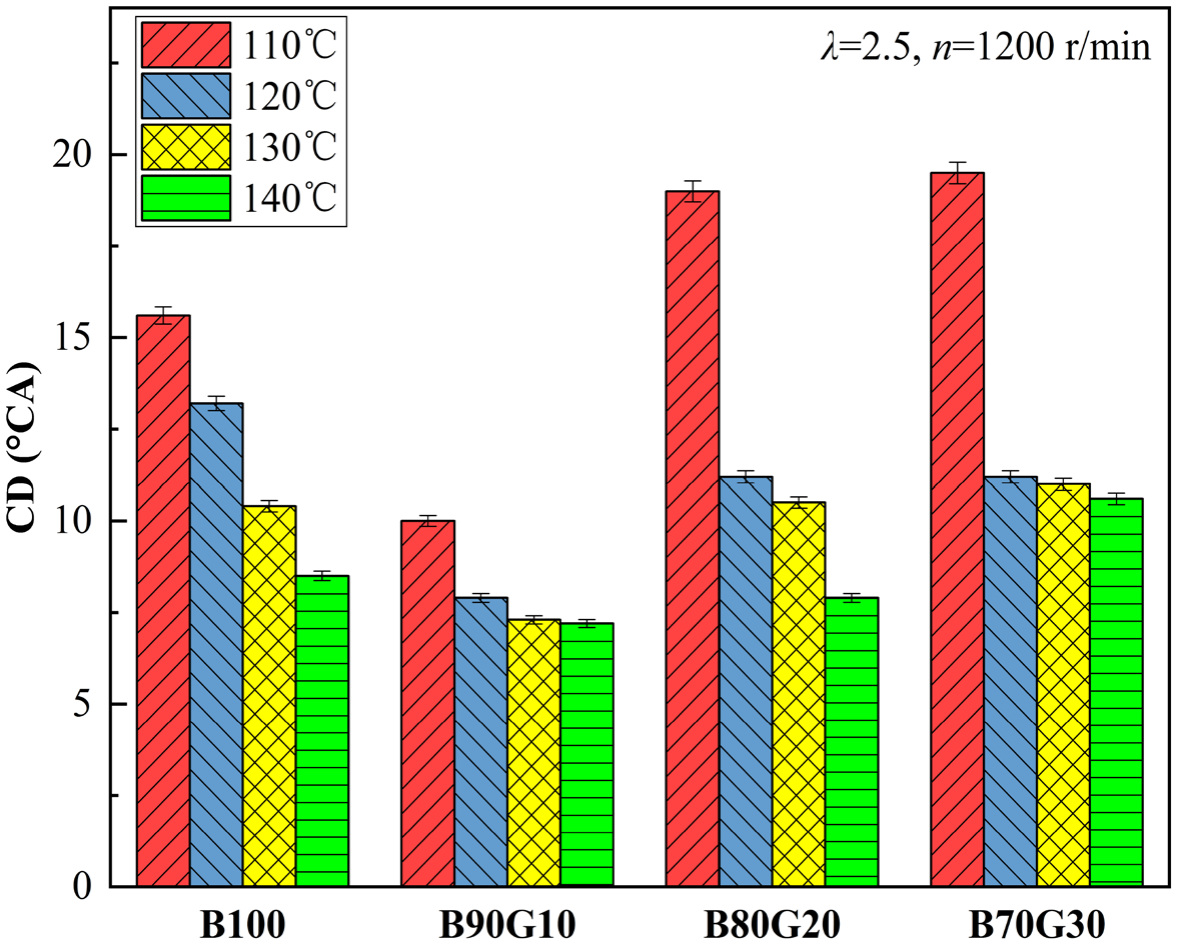
*CD* with different *T*_*in*_s.

Figure 15 shows the influence of *T*_*in*_ on *CD* of four test fuels. It can be seen that with the increase of *T*_*in*_, *CD*s of four fuels are continuously shortened. The main reason is similar to the above. With the increase of *T*_*in*_, mixture preparation is more uniform, activated molecules proportion increases, and the collision between molecules is exacerbated. The heat release is concentrated, which shortens the combustion time. It can also be seen that when *T*_*in*_ is 110 °C, *CD*s of B80G20 and B70G30 are significantly prolonged, mainly because *T*_*in*_ is lower than boiling point of n-butanol at this time, fuel atomization quality is poor, and the preparation of homogeneous mixture is blocked. In addition, n-butanol content in the blends decreases, and OH amount produced in low-temperature stage decreases, which is not conducive to fuel oxidative decomposition, which slows down chemical reaction rate and prolongs *CD*.

#### Cyclic variation

**Figure 16:**
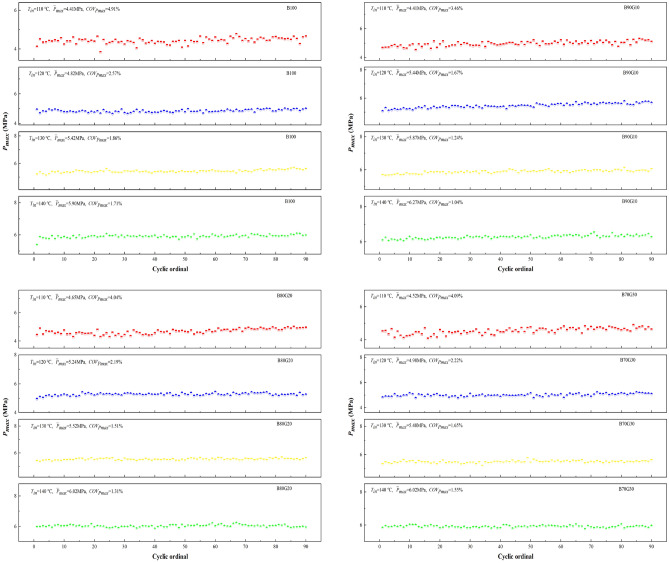
Cyclic variation with different *T*_*in*_s.

Figure 16 shows the influence of *T*_*in*_ on $\overline{P}_{max}$ and *COV*_*Pmax*_ of four test fuels. It can be seen that with the increase of *T*_*in*_, $\overline{P}_{max}$ of four fuels increases monotonically, while *COV*_*Pmax*_ is the opposite. For B90G10, with *T*_*in*_ is increased from 110 °C to 140 °C, $\overline{P}_{max}$ is increased from 4.41 MPa to 6.27 MPa, while *COV*_*Pmax*_ gradually is reduced from 3.46% to 1.04%. This is because the increase of *T*_*in*_ accelerates the reaction rate, shortens the combustion reaction time, and makes the peak pressure distribution relatively concentrated and *COV*_*Pmax*_ reduced. It can be seen that under the same *T*_*in*_, *COV*_*Pmax*_ of three blends are all smaller than that of B100, especially B90G10. The combustion stability of blends is better. Adding a small proportion of gasoline to n-butanol can improve combustion stability.

It can be seen from the previous analysis that after a small proportion of gasoline added to n-butanol, the preparation of combustible mixture is more uniform, the combustion exotherm is increased and concentrated. So in-cylinder temperature rises rapidly, and in-cylinder pressure rises accordingly. The continuous high-temperature environment can be maintained among engine working cycles, so that the PPCI engine combustion operation is stable. When added gasoline amount continues to increase, fuel activity decreases, number of active groups accumulated in low-temperature stage decreases, and heat release amount decreases, resulting in slower combustion reaction rate, lower in-cylinder temperature and pressure. As a result, the combustion stability is reduced, and therefore cyclic variation range is increased.

#### Emission characteristics

**Figure 17:**
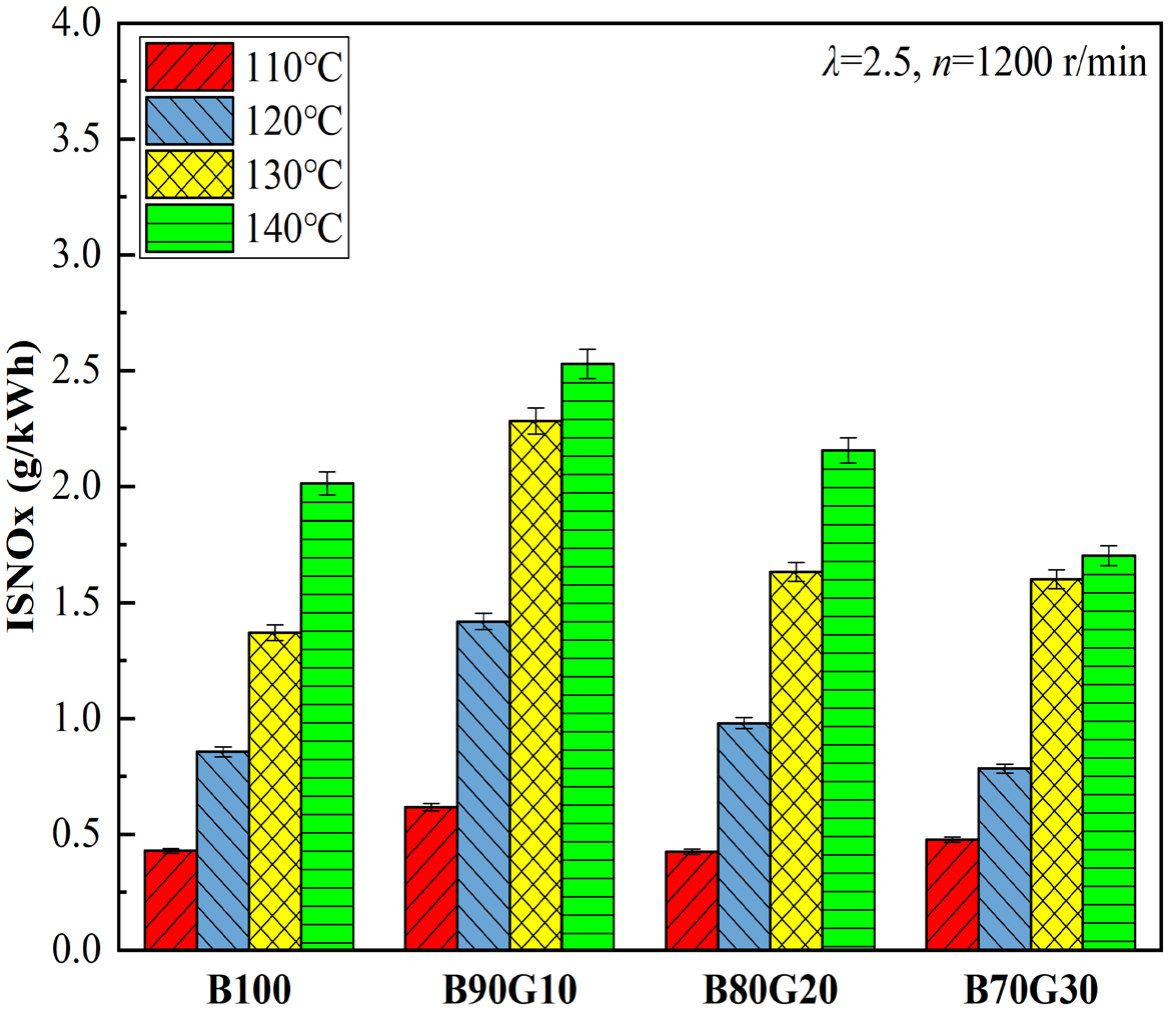
NOx emissions with different *T*_*in*_s.

Figure 17 shows the influence of *T*_*in*_ on NOx emissions of four test fuels. It can be seen that under different *T*_*in*_*s*, NOx emissions of four fuels are at ultra-low levels. This is because NOx emissions are highly related to peak combustion temperature and in-cylinder oxygen concentration. Although PPCI combustion uses partially homogeneous mixture and the combustion is in the oxygen-rich environment, in-cylinder temperatures of four fuels are less than 1800 K under different *T*_*in*_s, which is lower than the critical temperature for generating NOx, so their NOx emissions are low. It can be seen that under the same *T*_*in*_, NOx emissions of three blends are all higher than B100, especially B90G10. A small proportion of gasoline added to n-butanol may increase in-cylinder pressure and temperature, resulting in heat release more concentrated. So NOx emissions are increased. This is consistent with the previous analysis.

**Figure 18:**
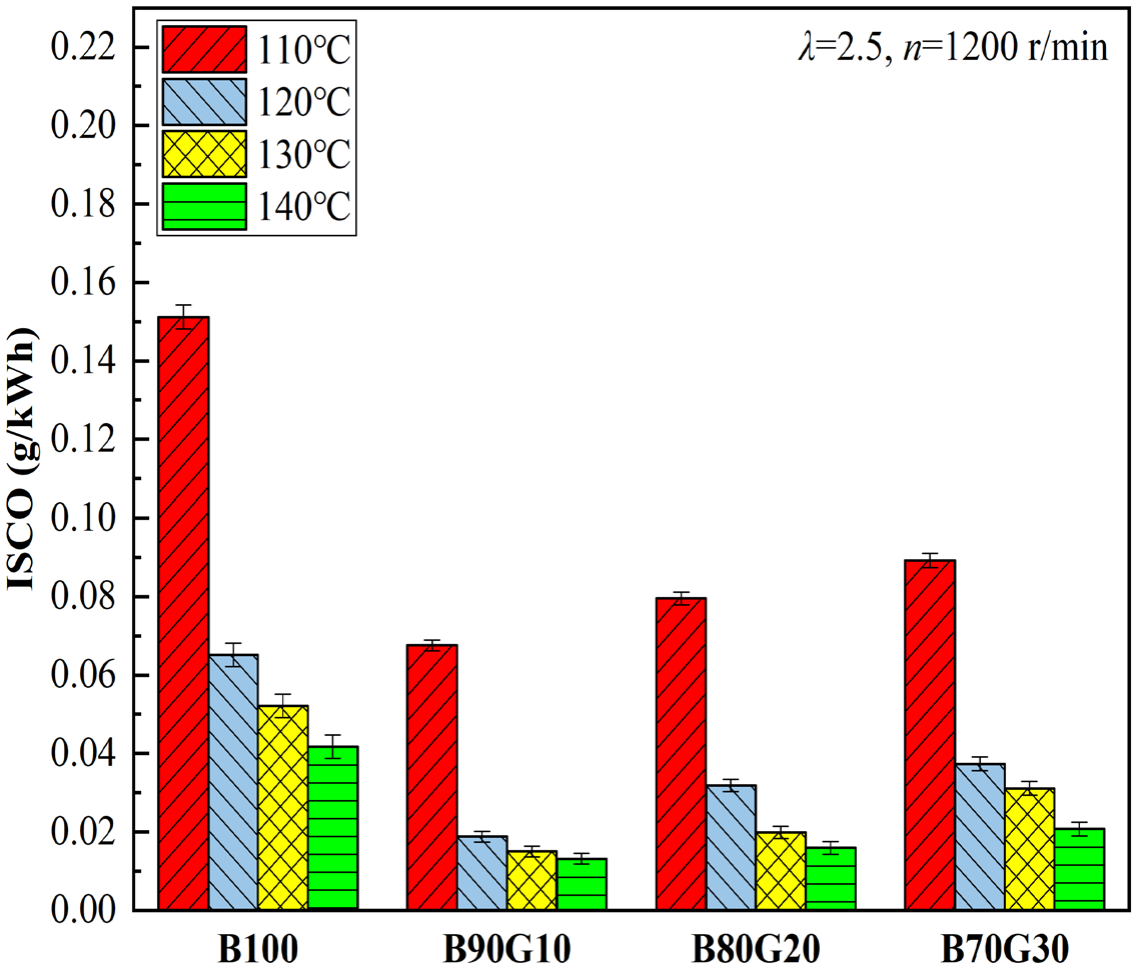
CO emissions with different *T*_*in*_s.

**Figure 19:**
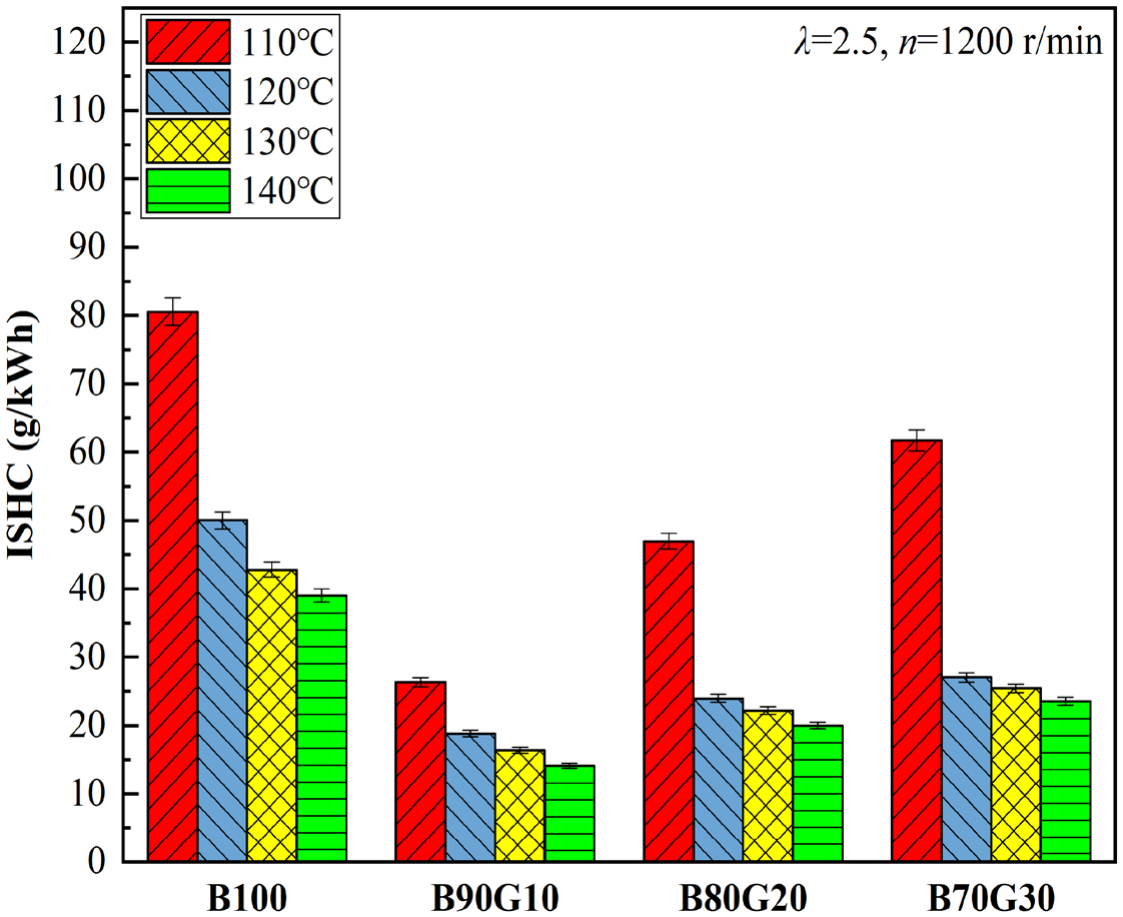
HC emissions with different *T*_*in*_s.

Figures 18 and 19 show the influence of *T*_*in*_ on CO and HC emissions of four test fuels, respectively. It can be seen that with the increase of *T*_*in*_, CO and HC emissions of four fuels show a monotonous decreasing trend. CO is an intermediate combustion product, and its generation is highly related to in-cylinder temperature. When the temperature is low, more CO is generated. With the increase of *T*_*in*_, mixture preparation is more uniform and the activity is significantly enhanced, which promotes the acceleration of chemical reaction rate and the combustion is more complete. In addition, with the increase of *T*_*in*_, in-cylinder temperature increases, which creates a favorable environment for the further CO oxidation. The generation condition of HC is similar to that of CO. The increase of in-cylinder temperature weakens cylinder-wall chilling effect and narrow-gap effect, which is beneficial to HC oxidation. In summary, with the increase of *T*_*in*_, CO and HC emissions decrease. It can be seen that under the same *T*_*in*_, CO and HC emissions of three blends are lower than B100, especially B90G10. Obviously, the combustion of n-butanol/gasoline blends may reduce CO and HC emissions. This is the opposite of the result of NOx emissions. From the previous analysis, it can be seen that after adding a small proportion gasoline to n-butanol, the prepared mixture is more uniform, and in-cylinder temperature is higher at the end of compression, which creates a favorable environment for CO oxidation. Simultaneously, cylinder-wall chilling effect and narrow-gap effect are weakened, and HC emissions are reduced. However, when the proportion of gasoline is further increased, the activity of fuel is reduced, and it is difficult to ignite. A large amount of unburned mixed gas enters piston-cylinder gap and fails to burn sufficiently. As the reaction progresses, CO_2_ cannot be produced, resulting in a gradual increase in CO production. At the same time, in-cylinder temperature and pressure may decrease, the combustion reaction rate may slow down, fuel may not burn sufficiently, cylinder-wall chilling effect and cylinder-gap effect may increase. It is not conducive to HC oxidation. Therefore, HC emissions may eventually rise as added gasoline proportion further increases.

## Conclusions

This paper is performed on PPCI engine fueled with n-butanol/gasoline blends to study the effects of excess-air coefficient (*λ*), intake temperature (*T*_*in*_*)* of different blends on the combustion and emission characteristics. The main conclusions are drawn as follows:

1. With the increase of gasoline blending ratio, the *P*_*max*_ and *HRR*_*max*_ both increase first and then decrease. Both *CA10* and *CA50* move forward earlier and then gradually lag, *CD* is shortened first and then extended, and *COV*_*Pmax*_ decreases first and then increases. When blended gasoline proportion is 10%, the *P*_*max*_, *T*_*max*_ and *HRR*_*max*_ reach peak values, while HC and CO emissions are the lowest.
2. For the four test fuels, with the increase of *T*_*in*_, the *P*_*max*_, *T*_*max*_ and *HRR*_*max*_ all show a monotonous increasing trend, and the corresponding crank angle positions are gradually move forward, *CA10* and *CA50* also are advanced, *CD* is shortened, and *COV*_*Pmax*_ is reduced. NOx emissions show an upward trend with the increase of *T*_*in*_, but the overall change is not large, all maintain at ultra-low levels, while HC and CO emissions gradually decline.
3. With the reduction of *λ*, the *P*_*max*_, *T*_*max*_ and *HRR*_*max*_ of four test fuels show a monotonically increasing trend, and the time of these parameter peaks appear is slightly ahead, but the change is not obvious, *CA10* basically has no change. The increase of mixture concentration is beneficial to reduce HC and CO emissions, while NOx emissions may increase slightly, but the overall changes are not large and all maintain at a very low level.
4. Under the corresponding *λ* and *T*_*in*_, compared with B100, the *P*_*max*_ and *HRR*_*max*_ of PPCI combustion fueled with n-butanol/gasoline blends increase, while *COV*_*Pmax*_, HC and CO emissions decrease. However, NOx emissions are increased.

Overall, B90G10 is the best blending fuel. *T*_*in*_ has the most significant effect on the n-butanol/gasoline PPCI engine. Within a certain temperature range, by controlling *T*_*in*_, the combustion process of the PPCI engine can be effectively controlled.

## Nomenclature

*CA10*: Crank angle corresponding to 10% of accumulated heat release (@°CA)
*CA50*: Crank angle corresponding to 50% of accumulated heat release (@°CA)
*CD*: Combustion duration (°CA)
*COV*_*Pmax*_: Coefficient of variation for peak in-cylinder pressure (%)
*HRR*: Heat release rate (kJ/°CA)
*n*: Engine speed (r/min)
*P*_*max*_: Peak in-cylinder pressure (MPa)
*T*_in_: Intake temperature (°C)
*T*_max_: The maximum in-cylinder temperature (K)
*λ*: Excess-air coefficient

## Abbreviations

CA: Crank angle
HCCI: Homogeneous charge compression ignition
PPCI: Partially Premixed Compression Ignition
